# A Sensitive Fluorescence Polarization Immunoassay for the Rapid Detection of Okadaic Acid in Environmental Waters

**DOI:** 10.3390/bios13040477

**Published:** 2023-04-16

**Authors:** Olga D. Hendrickson, Liliya I. Mukhametova, Elena A. Zvereva, Anatoly V. Zherdev, Sergei A. Eremin

**Affiliations:** 1 A. N. Bach Institute of Biochemistry, Research Center of Biotechnology, Russian Academy of Sciences, Leninsky Prospect 33, 119071 Moscow, Russia; odhendrick@gmail.com (O.D.H.); zverevaea@yandex.ru (E.A.Z.); zherdev@inbi.ras.ru (A.V.Z.); 2 Department of Chemical Enzymology, Faculty of Chemistry, M. V. Lomonosov Moscow State University, Leninskie Gory 1, 119991 Moscow, Russia; liliya106@mail.ru

**Keywords:** fluorescence polarization immunoassay, phycotoxins, okadaic acid, contamination, water safety

## Abstract

In this study, a homogeneous fluorescence polarization immunoassay (FPIA) for the detection of hazardous aquatic toxin okadaic acid (OA) contaminating environmental waters was for the first time developed. A conjugate of the analyte with a fluorophore based on a fluorescein derivative (tracer) was synthesized, and its interaction with specific anti-OA monoclonal antibodies (MAbs) was tested. A MAbs–tracer pair demonstrated highly affine immune binding (*KD* = 0.8 nM). Under optimal conditions, the limit of OA detection in the FPIA was 0.08 ng/mL (0.1 nM), and the working range of detectable concentrations was 0.4–72.5 ng/mL (0.5–90 nM). The developed FPIA was approbated for the determination of OA in real matrices: river water and seawater samples. No matrix effect of water was observed; therefore, no sample preparation was required before analysis. Due to this factor, the entire analytical procedure took less than 10 min. Using a compact portable fluorescence polarization analyzer enables the on-site testing of water samples. The developed analysis is very fast, easy to operate, and sensitive and can be extended to the determination of other aquatic toxins or low-molecular-weight water or food contaminants.

## 1. Introduction

Changes in the global climate occurring in recent decades, as well as the pollution caused by anthropogenic activities, have led to the warming of the surface waters of the world ocean [1]. This leads to massive algal blooms (“red tides”) becoming epidemic in some water bodies [2,3]. Microalgae (as well as cyanobacteria) are producers of aquatic toxins which are highly toxic for humans and animals [4,5]. They are highly likely to enter into fish and shellfish through food chains and accumulate in them. The pollution of environmental waters with aquatic toxins destroys marine and freshwater ecosystems, reduces the productivity of mariculture farms, damages tourism and recreational systems, and reduces biodiversity [2]. The consumption of contaminated fish and mollusks poses a great hazard to human health, causing intestinal disorders, and amnesic, hepatotoxic, and neuroparalytic symptoms, while chronic intake may provoke oncological diseases [6,7,8,9].

One of the aquatic toxins of top priority is okadaic acid (OA), produced by several species of dinoflagellates microalgae [10,11]. OA belongs to diarrheic shellfish poisoning (DSP) phycotoxins, which inhibit protein phosphatases possessing a variety of negative effects on animals and humans [12]. DSP provokes such acute symptoms as severe abdominal pain, diarrhea, nausea, vomiting, and others. Being a lipophilic compound, OA is mainly accumulated in the fatty tissues of shellfish and fish. Because it is resistant to high temperatures, the heat treatment of potentially contaminated water or food products will not lead to the destruction of this toxin. All these factors have led to the strict regulation of OA content in food products in many countries. Thus, the maximum residual level (MRL) of OA in shellfish tissues established in EU countries is 16 μg/kg [13].

The traditional techniques to control aquatic toxins, including OA, are mainly chromatographic methods with various types of detection, in particular, mass spectrometry [12,14]. These are undoubtedly reliable, accurate, and sensitive analytical methods that allow for the selective determination of the target analytes and their derivatives. However, their application requires complex equipment and qualified personnel and the analytical procedures are labor-intensive and time-consuming and include complex and long-term sample preparation before analysis. Therefore, these methods are promising as reference methods, are of little use for screening a large number of samples, and are ineffective for controlling the content of aquatic toxins outside stationary laboratories. In this aspect, immunoanalytical methods based on the specific and sensitive interaction of an analyte with antibodies have great potential.

The immunoassay of OA and other aquatic toxins includes, in particular, the heterogeneous enzyme-linked immunosorbent assay (ELISA) [15,16]. ELISA is a sensitive method that allows for testing dozens of samples simultaneously, but it cannot be considered fast because it is carried out in several stages (incubations) with microplate washing after each. A much faster immunoanalytical method is the immunochromatographic analysis (ICA), which allows for obtaining results in 10–15 min with ready-to-use test strips [17,18,19,20]. Among the shortcomings of the ICA, the time required for the assembly of a multi-membrane composite (test strips), including the application of specific immunoreagents on the membrane carriers, drying, cutting, etc., can be noted. Moreover, the ICA is often a qualitative (yes/no) or semi-quantitative technique, allowing for the visual determination of the presence of an analyte at concentrations above a cut-off level.

Polarization fluorescence immunoassays (FPIAs) are among the alternatives to these modes of heterogeneous immunoassays. The mechanism of fluorescence polarization (mP) is based on the fact that fluorophores emit light with different intensities along different polarization axes when irradiated with plane-polarized light. The FPIA is a homogeneous approach based on changing the mP of the reaction solution as a result of immune interactions [21,22]. The current trends in FPIAs and their application in bioassays are described in a recent review [23]. The traditional FPIA is based on the competitive binding of a free detectable analyte and an analyte labeled with a fluorescent molecule (the so-called tracer) with specific antibodies. The mP of a free tracer in a solution is low, and this increases when the tracer is bound with antibodies into an immunocomplex. Thus, the mP value of the reaction mixture reflects the ratio of the bound and free fractions of the tracer and is inversely proportional to the concentration of the analyzed compound. The degree of mP change depends on the label, the average lifetime of the molecule in the excited state, the molecular weight of the antigen, and the nature of the complex [21,23]. The average molecular weight of the free analyte and its complex with fluorophores should not exceed 20 kDa. In this regard, FPIAs are suitable for the detection of OA as a small-molecular-weight analyte (Mw = 805 Da). When choosing a label, a high fluorescence intensity (high quantum yield and extinction coefficient), the possibility of conjugation with an antigen, and stability are of decisive importance. These conditions are met by labels based on fluorescein and its derivatives [24]. In FPIAs, there is no need for the separation of bound and free fractions of reagents and washing; the assay duration is limited by the time needed for pipetting and commonly requires 5–10 min. Portable fluorescence polarization analyzers have now been developed that can be used for the on-site detection of analytes. Therefore, methodical simplicity, rapidness, accuracy, reproducibility, and sensitivity make the FPIA technique a promising approach for the rapid control of various analytes. The use of this method for the analysis of water samples, which requires no or minimal sample preparation, is especially of interest.

Currently, many studies have been published on the development of FPIAs using various toxicants, for example, mycotoxins, antibiotics, and pesticides [25,26,27,28]. The analytical application of this approach for the detection of aquatic toxins is limited to only two papers with the reported FPIAs using hepatotoxins microcystin-LR (MC-LR) and nodularin [29,30]. In these studies, the detection sensitivity was in the nanogram range. To the best of our knowledge, an FPIA-based test system for OA has not been reported thus far. In our investigation, a sensitive and rapid FPIA using phycotoxin OA is proposed for the first time. The developed assay was applied to analyze samples of environmental waters as a primary target for contamination with aquatic toxins.

## 2. Materials and Methods

### 2.1. Reagents and Materials

OA, domoic acid (DA), MC-LR, brevetoxin (BTX), soybean trypsin inhibitor (STI), bovine serum albumin (BSA), *N*,*N*′-dicyclohexylcarbodiimide (DCC), *N*-hydroxysuccinimide (NHS), *N*-(3-dimethylaminopropyl)-*N*′-ethyl-carbodiimide hydrochloride (EDC), fluorescein 5(6)-isothiocyanate (FITC), methanol, chloroform, dimethyl sulfoxide (DMSO), dimethyl formamide (DMFA), Triton X-100, and trimethylamine were acquired from Sigma-Aldrich (Saint Louis, MO, USA). Ethylenediamine dihydrochloride and 5-(aminomethyl) fluorescein hydrochloride (AMF) were acquired from ThermoFisher Scientific (Waltham, MA USA). A 3,3′,5,5′-tetramethylbenzidine dihydrochloride (TMB) substrate solution was purchased from Immunotekh (Moscow, Russia). Goat anti-mouse immunoglobulins labeled with horseradish peroxidase (GAMI–HRP) were obtained from Jackson Immuno Research Labs (West Grove, PA, USA). Monoclonal antibodies (MAbs) against OA (clone 7E1) were acquired from Santa Cruz Biotechnology (Dallas, TX, USA). All other compounds were analytically pure. For the ELISA test, polystyrene 96-well transparent microplates Costar 9018 from Corning Costar (Tewksbury, MA, USA) were used.

### 2.2. Synthesis of the Coating Antigens

OA–BSA and OA–STI conjugates were prepared according to the protocol described in [20]. During the synthesis, NHS (9 mg/mL, 200 μL), EDC (5 mg/mL, 200 μL), and OA (5 mg/mL, 400 μL), all in DMSO, were mixed and stirred for 30 min at room temperature (RT). Next, BSA or STI (800 μL both, 2.5 or 5 mg/mL, respectively, in 50 mM carbonate buffer, pH 9.5) was added dropwise to the indicated above solution of NHS, EDC, and OA in DMSO and incubated for 2 h at RT upon stirring. The reaction mixtures were dialyzed against a 50 mM K-phosphate buffer, pH 7.4, with 0.1 M sodium chloride (PBS) overnight at 4 °C. The obtained conjugates were stored at −18 °C.

### 2.3. ELISA of OA

As coating antigens, both OA–BSA and OA–STI were used. They were immobilized in microplate wells (1 μg/mL, 100 μL in PBS) for 2 h at 37 °C. The microplate was 4 times washed after each stage of ELISA with PBS containing 0.05% Triton X-100 (PBST). As a reaction medium and a diluent for immunorectants, PBST was further applied. For the determination of the antibody titer, MAb solutions (1 µg/mL–0.02 ng/mL) were added to the wells and incubated for 1 h at 37 °C. For the indirect competitive ELISA of OA, OA solutions (500 ng/mL–8 pg/mL, 50 µL) and anti-OA MAb solutions (7 ng/mL, 50 µL) were added to the wells and incubated as in the previous step. After that, GAMI–HRP (1:3000 dilution, 100 μL) was poured into the wells and similarly incubated. The HRP activity was measured based on the reaction with the TMB-based substrate. Briefly, 100 μL of its solution was added to each well, followed by a 10 min incubation at RT. The enzymatic reaction was terminated using 1 M H_2_SO_4_ (50 μL per well), and the absorbance at 450 nm (A_450_) was recorded using a Zenyth 3100 vertical photometer (Anthos Labtec Instruments, Wals, Austria).

### 2.4. Synthesis of Ethylenediamine Fluoresceinthiocarbamyl

Ethylenediamine fluoresceinthiocarbamyl (EDF) was synthesized based on the protocol described in [28]. Ethylenediamine dihydrochloride (200 mg, 1.5 µmol) was dissolved in 50 mL of methanol containing 0.5 mL of triethylamine. FITC (117 mg, 300 µmol) was dissolved in 10 mL of the same solvent and added dropwise to the stirred reaction mixture for 30 min. The resulting solution was incubated for 1 h at RT in the dark under stirring and then kept overnight in the same conditions. The orange precipitate formed as a result of the synthesis was filtered using a paper Whatman filter and then washed with 10 mL of methanol. The final product was dried at RT in the dark.

### 2.5. Synthesis of OA–EDF and OA–AMF

The procedure of tracer synthesis was similar to that described in [31]. The OA:DCC:NHS molar ratio during synthesis was 1:2:2. DCC (0.25 mg) and NHS (0.15 mg) were dissolved in 200 µL of DMFA. Then, OA (0.5 mg) was added, and the solution was incubated for 18 h at RT. The reaction mixture was centrifuged at 10,000 rpm for 3 min, and the supernatant was collected. After that, AMF or EDF (both 0.25 mg) and 10 µL of trimethylamine were added to the supernatant and incubated for 24 h in the dark. The reaction mixture was then purified via thin-layer chromatography (TLC) in chloroform–methanol, at a 4:1 ratio. All yellow-colored bands were collected and dissolved in methanol.

The concentration of the tracers was calculated from the measured absorbance at 492 nm in a 50 mM carbonate buffer, pH 9.0, using the following formula: c = A/ε × l, where A is an absorbance, ε is the extinction coefficient (8.7 × 10^4^ L/mol × cm), and l is the optical path length [32]. The stability of the obtained tracers under storage was confirmed using repeated TLC. The tracer structure was studied using high-resolution tandem mass spectrometry coupled with high-performance liquid chromatography (see Supplementary Materials).

### 2.6. FPIA of OA

#### 2.6.1. Choice of Tracer and MAbs Working Concentrations

To select the working concentration of the tracer, a series of dilutions (0.1–20 nM, 1 mL) in a 25 mM borate buffer, pH 8.5 (BB), was prepared in the test tubes. To select the working concentration of MAbs, a series of dilutions (0.3–22 nM, 0.5 mL) in BB were mixed with the tracer (5 nM, 0.5 mL) in test tubes and incubated for 5 min. Then, fluorescence intensity and mP were measured using a Sentry 200 portable fluorescence polarization reader (Ellie LLC, Germantown, WI, USA).

#### 2.6.2. Kinetics of MAbs—Tracer Interaction

MAbs (5 nM) and the OA–EDF tracer (5 nM) in BB were mixed in a test tube (0.5 mL both). The mP values were measured at 25 °C for 1 h with a 1 min interval.

#### 2.6.3. Competitive FPIA

To a series of OA solutions (1000–1 ng/mL, 0.5 mL), a tracer solution (5 nM, 0.5 mL) was added and thoroughly vortexed. Then, MAbs (50 nM, 50 µL) were added. After 5 min incubation of the reaction mixture, mP was measured. Upon testing the real samples, water was mixed with the tracer instead of OA solutions.

### 2.7. Evaluation of the Assay Results

The plots of A_450_ or mP (y) versus OA concentrations (x) were drawn and fitted to a four-parameter logistic function using the Origin software (OriginLab, Northampton, MA, USA). The OA LODs, cutoffs, and working ranges were evaluated as described in [33,34]. To determine the titer of MAbs, a plot of A_450_ versus the MAb concentrations (x) was drawn. The titer was calculated as a concentration providing reliable difference from the background A_450_ value in accordance with the “3 SD” criteria [35].

### 2.8. Pretreatment of Seawater Samples

Seawater samples were collected from the Aegean Sea (Fethiye, Turkey), the Mediterranean Sea (Side, Turkey), the Barents Sea (Murmansk, Russia), and the Volkhov River (Nizhniy Novgorod, Russia) and stored at 4 °C. Before the FPIA, water samples were spiked with OA and analyzed without subsequent sample preparation.

## 3. Results and Discussion

### 3.1. Obtaining and Characterization of the Specific Reagents

#### 3.1.1. Hapten–Protein Conjugates and MAbs

**Figure 1:**
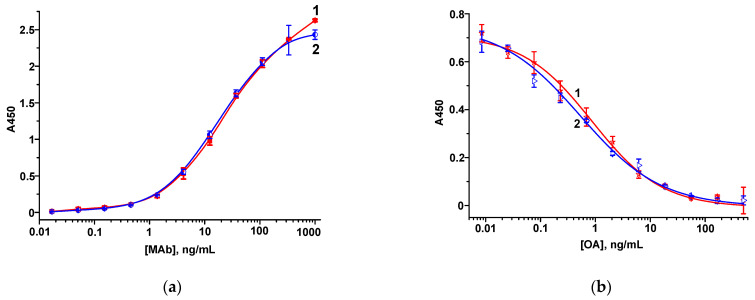
Dependence of A_450_ on the concentration of MAbs (**a**) and calibration curves of OA (**b**) in the ELISA with immobilized OA–BSA (1) and OA–STI (2) (*n* = 3).

As a receptor for OA, commercial anti-OA MAbs were used. Before performing the FPIA, the preparation of MAbs involved an indirect ELISA. As coating antigens for the ELISA, two OA–protein conjugates were synthesized using the carbodiimide technique and characterized via spectrophotometry. The peaks representative of hapten and protein carriers were observed (data not shown), which provided evidence of successive conjugation. The titer of MAbs was considered the minimal concentration of antibodies ensuring a reliably detected interaction with the immobilized antigen (see Section 2.7). The calculated titer was 1.7 ng/mL, independent of the immobilized hapten–protein conjugate. In the competitive ELISA, OA was found to have LODs of 0.04 ng/mL and 0.01 ng/mL with the immobilization of OA–BSA and OA–STI, respectively (Figure 1). The high analytical characteristics ensured by the antigen–antibody pair in the heterogeneous assay allow for the development of a homogeneous FPIA.

#### 3.1.2. Fluorescent Tracers

**Figure 2:**
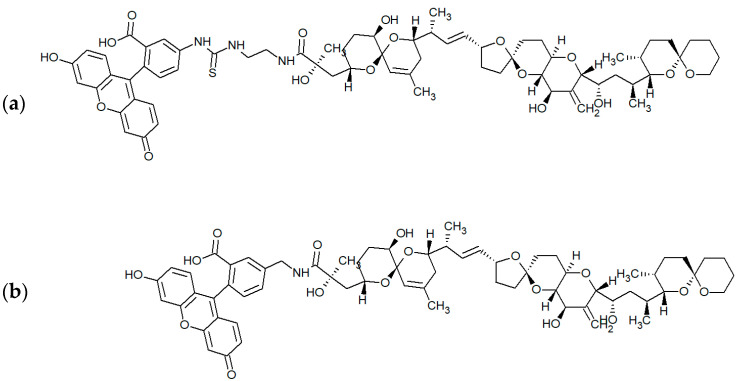
Structures of OA–EDF (**a**) and OA–AMF (**b**) tracers.

To implement the FPIA, it is necessary to obtain an antigen labeled with a fluorescent marker (tracer). In this study, two derivatives of fluorescein, i.e., EDF and AMF, were investigated as fluorophores. The presence of functional amino groups in the structure of the fluorophore allows for a simple procedure of conjugation with OA containing a carboxyl group with the formation of an amide bond (the structures of OA–EDF and OA–AMF tracers are shown in Figure 2).

**Figure 3:**
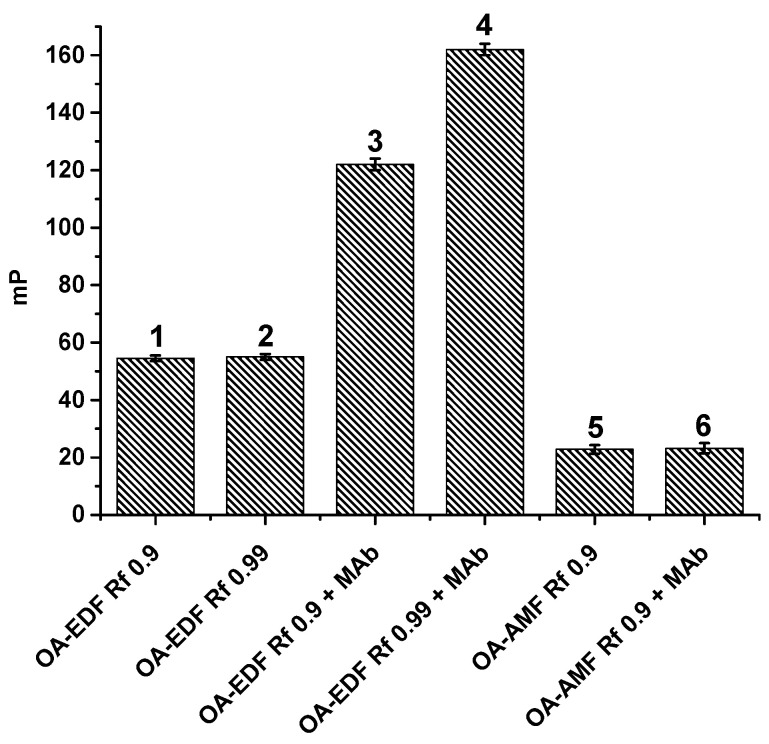
Registered mP values for solutions of free tracers and after their interaction with MAbs (*n* = 3).

AMF was commercially prepared and contained an existing NH_2_ group, whereas EDF was synthesized based on FITC through which an amino-group-containing derivative was obtained. OA–EDF and OA–AMF conjugates were synthesized via carbodiimide activation and purified using TLC. As a result, two fractions were obtained for each tracer with retention factors (*Rf*) of 0.6 and 0.9. Fractions with *Rf* 0.6 corresponded to the unreacted fluorophores, which showed the same mobility when they were applied to the plate as a control. Therefore, we excluded these derivatives from further consideration. The remaining fractions were tested for interaction with MAbs in the FPIA. Before this procedure, working dilutions of tracers were selected. For this purpose, a series of dilutions of OA–EDF and OA–AMF conjugates were prepared in buffer solutions, and the intensity (I) and mP values were measured for each dilution. A working dilution of the tracer was chosen at the point of a sharp increase in the mP value so that the fluorescence intensity of such a solution was at least 20 times higher than the intensity of the background signal (measured in the buffer solution). According to the obtained data, the working concentrations of all tracers were 2.5 nM. At a given concentration, the tracers yielded the optimal signal-to-noise ratio and, consequently, a stable mP value. Next, tracers at the selected concentration were added to MAbs at a fixed concentration, and mP was recorded. It was found that the OA–AMF tracer did not interact with MAbs (columns 5 and 6 in Figure 3), and only the OA–EDF tracer with *Rf* 0.9 demonstrated the specific binding together with an increase in the mP value (compare columns 1 and 3 in Figure 3). This may be explained by the shorter spacer between OA and AMF than that between OA and EDF (Figure 2). Because of this difference, in the case of AMF fluorophore, part of the tracer may interfere with OA–antibody interaction, especially if the epitope recognized by the antibodies is not opposite of the fluorescein conjugation point.

The fluorescence intensity and fluorescence polarization of the tracer solution did not reliably alter during its storage for at least 6 months. There were also no reliable changes in the mP increase in the experiments with the interaction of the tracer and antibodies under fixed concentrations during this time.

It is known that sensitive FPIAs require the use of high-quality immunoreagents. Therefore, the selected OA–EDF tracer was additionally purified via TLC under the same conditions. The isolated compound was characterized by *Rf* 0.99; its working concentration for the FPIA was selected as described above. This purified tracer was also tested for antibody binding (column 4 in Figure 3). As can be seen, the mP values of the free tracer did not change after additional purification (~55 units for both preparations; compare columns 1 and 2 in Figure 3) but noticeably increased upon binding to MAbs (by approximately 40 units when using an additionally purified tracer; compare columns 3 and 4 in Figure 3). Thus, this OA–EDF tracer (*Rf* 0.99) was used in further FPIAs. Its structure was confirmed via high-resolution tandem mass spectrometry coupled with high-performance liquid chromatography (see Figure S1).

### 3.2. FPIA of OA

**Figure 4:**
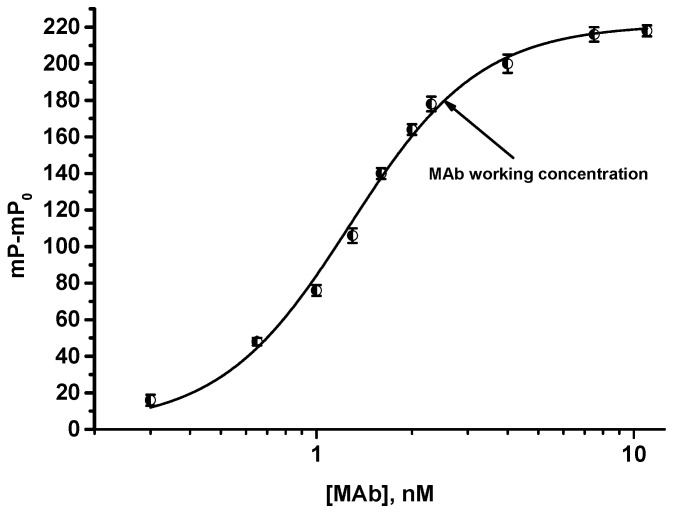
Dependence of changing the fluorescence polarization signal (mP-mP_0_) on the concentration of MAbs after the interaction with tracer (2.5 nM), *n* = 3.

To select the working concentration of MAbs for the competitive FPIA, the dependence of mP on the concentration of MAbs after their interaction with the tracer was analyzed (Figure 4). The selected concentration has to meet two requirements: to ensure a high analytical signal for accurate and reproducible analysis, on the one hand, and to allow for the achievement of the sensitive OA detection, on the other. These requirements are fulfilled at a concentration of antibodies that provide 50–80% binding with antigens [28,36]. Lower concentrations of antibodies cause a significant reduction in the detected signal and worse accuracy of the assay, whereas their higher concentrations limit the sensitive competitive detection of free antigens. Taking these reasons into account, the MAb concentration providing 80% binding was selected, namely 2.5 nM (Figure 4). As shown in Figure 4, the saturation point corresponded to an antibody concentration of approximately 3 nM. Given the fact that the tracer concentration was 2.5 nM, and taking into account the bivalence of antibodies (i.e., each antibody molecule can interact with two tracer molecules), the expected saturation point was somewhat lower and corresponded to an antibody concentration of approximately 1.5 nM. The observed effect may be explained by the partial loss of immune reactivity due to the local inactivation of antigen-binding sites.

To quantitatively characterize the antibody–tracer interaction, we used the procedure proposed in [37] and adopted it to include antigen-binding sites (ABS) of bivalent MAb of IgG type. The interaction of ABS with OA–EDF is expressed using Equation (1). Because one MAb molecule can bind two antigen molecules, in the calculations, we consider the concentration of ABS to be 2 times higher than the MAb concentration:

[ABS] + [OA − EDF] = [ABS − OA − EDF].(1)

The equilibrium dissociation constant (*KD*) is expressed as follows:

(2) $$KD=\frac{\left[ABS\right][OA-EDF]}{\left[ABS\times OA-EDF\right]}.$$

To estimate the ABS content, we consider the total concentrations of interacting compounds: ABS_T_ and OA–EDF_T_. The following equation is obtained:

(3) $$[\mathrm{ABS}]=[{\mathrm{ABS}}_{T}]-[ABS-OA-EDF].$$

Therefore, the proportion of the bound fraction is as follows:

(4) $$F_{bound}=\frac{\left[ABS-OA-EDF\right]}{\left[{ABS}_{T}\right]}=\frac{mP-{mP}_{0}}{\left({mP}_{bound}-mP\right)Q+(mP-{mP}_{0})},$$

where mP_0_ is the mP of free fluorescently labeled antigen, mP_bound_ is the mP of the antibody–antigen complex upon binding, and Q is the ratio of the fluorescence intensities of the bound and free antigens. When measuring the mP of the samples, the intensities of bound forms did not change (Q = 1).

Therefore,

(5) $$F_{bound}=\frac{((a)-\sqrt{\left(({a)}^{2}-4[{ABS}_{T}][{OA-EDF}_{T}]\right)}}{2[{ABS}_{T}]}=\frac{mP-{mP}_{0}}{\left({mP}_{bound}-mP\right)+(mP-{mP}_{0})},$$

where a = *KD* + [ABS_T_] + [OA − EDF_T_].

*KD* was calculated using Equation (6):

(6) $$mP-mP_{0}=\left(mPb-mP_{0}\right)\times \left(\frac{a-\sqrt{{\left(a\right)}^{2}-4*[{ABS}_{T}][{OA-EDF}_{T}]}}{4}\right).$$

Taking into account the bivalence of antibodies, the calculated *KD* was 0.8 nM.

**Figure 5:**
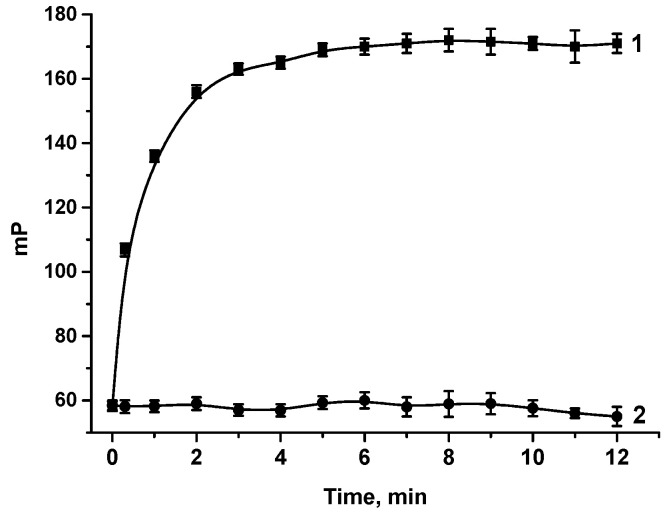
Kinetics of the tracer interaction with specific (1) and nonspecific (2) MAbs.

To select the duration of the FPIA, the binding kinetics was studied, for which anti-OA MAbs and the tracer were incubated at working concentrations, and mP values were measured within 1 h with a 1 min periodicity. The kinetic interaction curve is shown in Figure 5 (curve 1). The obtained data demonstrate a rapid increase in mP and achieving equilibrium in 5 min (mP reaches a plateau). Thus, the reaction mixture was subsequently incubated for 5 min in the competitive FPIA. For the control experiment, the kinetics of OA–EDF binding with antibodies against another phycotoxin, DA, was evaluated. It was found that upon the incubation of the tracer with anti-DA antibodies, the mP value remained unchanged throughout the entire incubation period, which confirms the absence of nonspecific binding (curve 2 in Figure 5).

**Figure 6:**
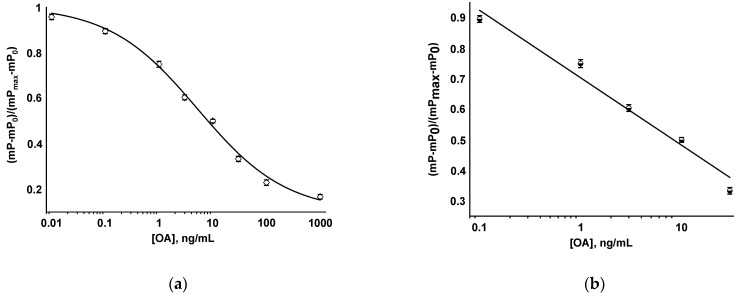
Calibration curve of OA in FPIA (**a**) and its linear range (**b**).

Under the selected conditions, a competitive FPIA was implemented, based on the competition between the detected antigen and the tracer for the limited number of antibody binding sites. The higher the concentration of the detected analyte in the sample, the more the free-labeled antigen remained in the reaction mixture and, accordingly, the lower the measured mP value. The obtained OA calibration curve and its linear range are presented in Figure 6.

Using the developed FPIA, OA concentration was determined with the LOD of 0.08 ng/mL (0.1 nM), and the working range of detectable concentrations was 0.4–72.5 ng/mL (0.5–90 nM). The duration of measurements was 5 min.

### 3.3. Study of the Assay Specificity

The specificity of the developed test system was assessed by testing the cross-reactivity (CR) to the relevant toxins that may contaminate environmental waters. The CR (%) was calculated as IC50_CR_/IC50_OA_ × 100, where IC50_CR_ and IC50_OA_ are the concentrations of the cross-reactant and OA causing 50% inhibition of MAb binding with the tracer. Among the different cross-reactants, DA was used as a neurotoxin, which belongs to amnestic shellfish-poisoning toxins and, similar to OA, may reach significant concentrations in reservoirs during water bloom. Moreover, the hazardous neurotoxin BTX and hepatotoxin MC-LR, which can pollute both fresh waters and seawater, were detected. It was found that the mP did not change even for such a high concentration of the tested competitors as 1000 ng/mL. So the cross-reactivities to DA, BTX, and MC-LR could not be quantified and were estimated as less than 0.1% for all three compounds. This indicated that the developed FPIA had high specificity only towards OA.

### 3.4. Determination of OA in Water

The developed FPIA was performed for the determination of OA in environmental waters. The preliminary testing of all the studied samples using commercial OA ELISA kits (EuroProxima, Arnhem, The Netherlands) revealed no OA content. It should be noted that seawater and river water are complex matrices with components (salts and other impurities) that may affect the results of the homogeneous FPIA. In our case, the matrix effect was not observed, thus highlighting an essential advantage of this analytical method. Water samples were spiked with OA at the concentrations selected from the working range and analyzed using the FPIA. The resulting recovery values are presented in Table 1.

The obtained data show that using the developed FPIA, 90–115% of OA was detected in river water and seawater.

### 3.5. Comparison of the Obtained Results with Other Studies

As mentioned above, the FPIA of OA is not described in the literature, and the immunoassay of this toxin is mainly focused on ELISAs and ICAs (in this context, we did not consider a few studies on biosensors and microfluidic approaches). Therefore, we compared the analytical performance of the developed FPIA with that of the ELISA and ICA. Table 2 summarizes data on the sensitivity, assay duration, and types of matrices tested by the proposed analyses in different studies.

In general, LODs of OA are lower in ELISAs, approximately reaching picogram values [38,39,40,41,42,43,44,45,46,47]. In ICAs [44,45,46,47,48,49], including multiparametric ones [18,20,50], the sensitivity of the determination is somewhat worse (up to the nanogram concentration range), and improvements in this method were achieved with amplification approaches aimed at increasing analytical signal [19,51]. The duration of the analysis in the case of ICA is 10–40 min. For ELISAs, the assay time is usually not indicated in the papers, but taking into account that each incubation step continues for 45–60 min, and the whole analytical procedure includes 2–3 incubation steps as well as washing and pipetting, it can be concluded that the common ELISA requires several hours. In comparison with the presented ELISA and ICA characteristics, those of the FPIA developed in this study proved very promising. Thus, the achieved LOD (0.08 ng/mL, 0.1 nM) is comparable to or even lower than that in the ELISA or the enhanced ICA [51]. Because there is no need for the separation of bound and unbound immunoreagents, the duration of the FPIA is only 5 min, which is even shorter than all the rapid ICA-based test systems considered. In addition, the FPIA does not require sample preparation of the liquid matrix, unlike the ICA of the same matrices [19,45]. The preparation for the analysis requires only the synthesis of a tracer and does not include any preliminary stages such as the sorption of coating antigens (similar to the microplate-based ELISA) or the application of the reagents on membrane carriers (similar to the ICA). In addition, the portable mP analyzer allows detection in out-of-laboratory conditions.

Therefore, FPIAs can serve as effective alternatives not only to traditional ELISAs but also to rapid immunochromatographic tests for the fast, simple, sensitive, and reproducible analysis of OA and other low-molecular-weight contaminants in food and water. In terms of future research, the next step in the validation of this assay is its application in naturally contaminated samples.

## 4. Conclusions

A highly sensitive FPIA was designed for the detection of phycotoxin OA. Due to the homogeneous format, the assay can be performed within 5 min via a simple incubation procedure of the analyte, specific antibodies, and the tracer. The achieved LOD for OA was 0.08 ng/mL (0.1 nM). The use of a portable analyzer enables the out-of-laboratory control of OA content. The developed FPIA was successfully applied for the determination of OA in river and seawater samples, with recovery coefficients of 90–115%. This approach is recommended as an analytical tool for the fast and reliable detection of aquatic toxins as well as other toxicants.

## Figures and Tables

**Table 1:** Recoveries of OA from river water and seawater (*n* = 3).

Water Sample	Sampling Location	Added OA Concentration, ng/mL	Detected OA Concentration, ng/mL	Recovery, %
1	The Volkhov River	1	0.9 ± 0.1	90.0 ± 2.0
		3	3.2 ± 0.2	107.0 ± 1.5
		20	21.2 ± 0.2	106.0 ± 1.6
2	The Barents Sea	1	1.1 ± 0.1	110.0 ± 2.0
		3	3.1 ± 0.2	103.0 ± 3.1
		20	19.5 ± 0.2	97.5 ± 1.2
3	The Mediterranean Sea	1	1.1 ± 0.1	112.0 ± 2.0
		3	3.1 ± 0.2	103.0 ± 1.5
		20	22.2 ± 0.2	111.0 ± 1.6
4	The Aegean Sea	1	1.2 ± 0.1	115.0 ± 2.0
		3	2.8 ± 0.2	93.3 ± 1.2
		20	21.6 ± 0.2	108.0 ± 1.0

**Table 2:** Studies on the ELISA, ICA, and FPIA of OA.

N	Assay Format	Assay Performance	Assay Duration, min	Matrix	Reference
1	ELISA	*IC50* = 4.4 ng/mL	n.i. *	Mollusks	[15]
2	ELISA with magnetic beads	LOD = 0.35 ng/mL	n.i.	Shellfish	[16]
3	Enzyme-linked immunosensor based on super-paramagnetic nanobeads	LOD = 0.38 ng/mL	60	Mussels	[38]
4	Smartphone-assisted microarray immunosensor based on ELISA	LOD = 0.02 ng/mL	n.i.	Shellfish	[39]
5	ELISA	*IC50* = 0.15 ng/mL	n.i.	Oysters and green mussels	[40]
6	Chemiluminescent ELISA	LOD = 0.012 ng/mL	n.i.	Buffer	[41]
7	Chemiluminescent ELISA	LOD = 0.175 ng/g	n.i.	Shellfish	[42]
8	Enhanced ELISA with nanozymes	LOD = 0.04 ng/mL	n.i.	Oysters, mussels, and clams	[43]
9	ELISA/ICA	*IC50* = 6.4/2.4 ng/mL	n.i./30	Shellfish	[44]
10	ELISA/ICA	*IC50* = 0.077 ng/mL/ LOD = 5 ng/mL	n.i./10	Clams, scallops, mussels, and oysters	[45]
11	ELISA/ICA	LOD = 0.012/0.1 ng/mL	n.i./5	Shellfish	[46]
12	ELISA/ICA	LOD = 0.023/5 ng/mL	n.i./10	Mussels	[47]
13	ICA	LOD = 0.45 ng/mL	40	Shellfish	[48]
14	ICA	LOD = 50 ng/mL	10	Shellfish	[49]
15	ICA	LOD = 25 µg/kg	20	Shellfish	[17]
16	Double ICA of OA and tetrodotoxin	LOD = 0.75 ng/mL	10	Clams	[18]
17	Double ICA of OA and DA	LOD/cutoff = 0.1/2.5 ng/mL	18	Seawater, octopuses, mussels, tiger shrimps, crabs, whelks, and scallops	[20]
18	Triple ICA of OA, DA, and MC-LR	0.1/2.0 ng/mL	18	Seawater, river water, and fish	[50]
19	Enhanced ICA with nanozyme	LOD/cutoff = 0.5/10 ng/mL	20	Seawater, river water, and fish	[19]
20	Enhanced with cascade amplification	LOD/cutoff = 0.03/1 ng/mL	43	Seawater, fish, tiger shrimps, and scallops	[51]
21	FPIA	LOD = 0.08 ng/mL	5	Environmental waters	This study

* Not indicated.

## Data Availability

The original data presented in this study are included in the article; further inquiries can be directed to the corresponding author.

## Supplementary Materials

The following supporting information can be downloaded at: https://www.mdpi.com/article/10.3390/bios13040477/s1. Figure S1. Mass spectra of OA–EDF tracer.
