# Supplementary material for: A Sensitive Fluorescence Polarization Immunoassay for the Rapid Detection of Okadaic Acid in Environmental Waters
Source: Biosensors (Basel). 2023 Apr 16;13(4):477. doi: 10.3390/bios13040477 (PMC10136290; doi:10.3390/bios13040477)
Supplement: Supplementary file 1 [file biosensors-13-00477-s001.zip › biosensors-2300649-supplementary.pdf]

biosensors

Supplementary Material

A Sensitive Fluorescence Polarization Immunoassay for the Rapid Detection of Okadaic Acid in Environmental Waters

Olga D. Hendrickson [1], Liliya I. Mukhametova [2], Elena A. Zvereva [1], Anatoly V. Zherdev [1], and Sergei A. Eremin [1,2,*]

[1] A. N. Bach Institute of Biochemistry, Research Center of Biotechnology, Russian Academy of Sciences, Leninsky Prospect 33, 119071 Moscow, Russia; odhendrick@gmail.com (O.D.H.); zverevaea@yandex.ru (E.A.Z.); zherdev@inbi.ras.ru (A.V.Z.)
[2] Department of Chemical Enzymology, Faculty of Chemistry, M. V. Lomonosov Moscow State University, Leninskie Gory 1, 119991 Moscow, Russia; liliya106@mail.ru
* Correspondence: saeremin@gmail.com; Tel.: +7-916-5127654

Mass spectra were obtained using a Q-Exactive tandem mass spectrometer coupled to an Ultimate 3000 high-performance liquid chromatograph; samples were ionized via electrospray in a HESI-II ionization source (Thermo Scientific, Waltham, MA USA). The mass spectrum of the first order, which was obtained in the mode of registration of positively charged ions, is presented below.

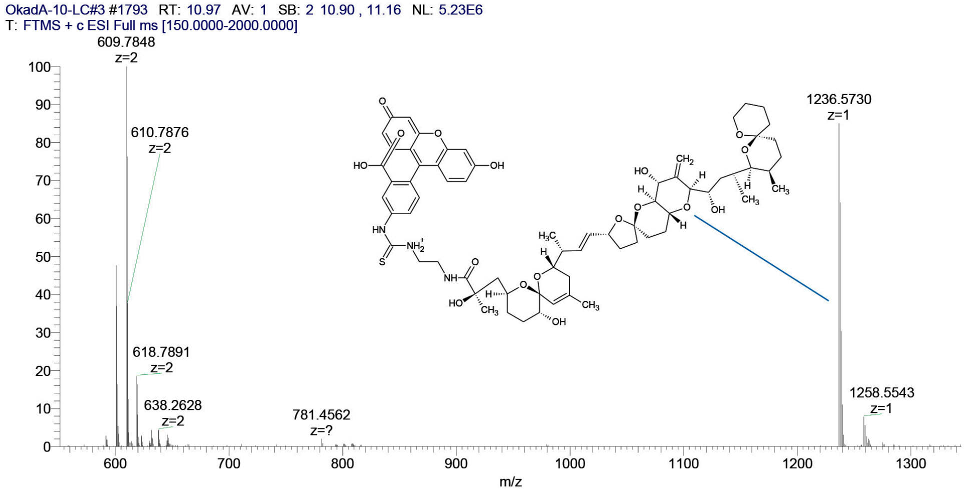

Figure S1. Mass spectra of OA–EDF tracer.
